# Sex Differences in Risk Factors for Transient Ischemic Attack in a Chinese Population

**DOI:** 10.3389/fneur.2021.615399

**Published:** 2021-05-06

**Authors:** Wendi Wang, Pei Sun, Fengyue Han, Chuanqiang Qu

**Affiliations:** ^1^Neurology Department, Shandong Provincial Hospital Affiliated to Shandong First Medical University, Jinan, China; ^2^Neurology Department, Shandong Provincial Hospital, Cheeloo College of Medicine, Shandong University, Jinan, China

**Keywords:** distributional differences, Eastern China, transient ischemic attack population, risk factors, stroke, sex differences

## Abstract

**Introduction:** This study aimed to collect and evaluate basic information of a stroke screening population in eastern China and to compare distribution differences in risk factors between males and females in a transient ischemic attack (TIA) population.

**Methods:** A standardization of the risk factors for stroke was performed according to an implementation plan of stroke in a high-risk population screening and intervention project in Shandong Province. Of the 231,289 residents, 8,603 patients with a previous TIA were identified and risk factors in this cohort were analyzed for sex differences.

**Results:** In our initial cohort of 231,289 residents, we found 3,390 men and 5,213 women with TIA, accounting for a prevalence of 3.1 and 4.2%, respectively. Risk factors for TIA were hypertension, atrial fibrillation, diabetes, smoking, lack of exercise, overweight, and family history of stroke. In our TIA cohort, we found that the prevalence of smoking was significantly higher in men (41.3%) compared with that found in women (4.2%). Further, hypertension (58.8 vs. 55.5%) and family history of stroke (22.3 vs. 20.0%) were more prevalent in men compared with women, whereas atrial fibrillation (AF) (14.7 vs. 16.4%), diabetes (11.1 vs. 13.2%), lack of exercise (27.2 vs. 28.0%), and overweight (29.5 vs. 35.7%) were less prevalent.

**Conclusions:** In our TIA cohort from eastern China, we found significant sex differences for the risk factors of hypertension, atrial fibrillation, smoking, diabetes, and overweight.

## Introduction

Stroke is a severe disease of global significance with a high disability rate and high incidence (1). In the past, transient ischemic attack (TIA) was defined as any focal ischemic event with symptoms lasting 24 h. Stroke 2009 mated that a revision of TIA definitions to“a transient episode of neurological dysfunction caused by focal brain, spinal cord, or retinal ischemia without acute infarction” (2). As TIA is a significant predictor of subsequent ischemic stroke (3), it has been hypothesized that there may be sex differences in the incidence of TIA over time. Further, because TIA constitutes a major risk factor for stroke, patients with TIA are an important group for secondary intervention. The fact that diabetes mellitus, atrial fibrillation, and smoking are more common in individuals with TIA compared with the general population suggests that these factors are risk factors for TIA (4). In addition, as a risk factor and precursor of cerebral infarction, TIA is of high clinical value. Therefore, understanding the risk factors of TIA in the context of stroke risk will enable timely and effective implementation of preventive countermeasures, thereby reducing the probability that a TIA event leads to infarction. There are also substantial sex differences in age-adjusted stroke incidence and stroke prevalence as well as in the prevalence and risk of various cardiometabolic factors, including hypertension, atrial fibrillation, diabetes mellitus, and smoking (5). An improved understanding of these differences is needed to ensure that stroke prevention strategies are effective for both women and men.

It has been reported that sex differences contribute to differences in the prevalence and/or control of TIA risk factors (5). One study reported that ischemic stroke incidence has declined over time for men but not women. In males, they found that incidence decreased from 153 per 100,000 (95% confidence interval [CI] 139–167) in 1993 and 1994 to 117 per 100,000 (95% CI 107–128) in 2010 (*P* < 0.05 for trend test), with no significant change observed among females for the same period [107 vs. 102 per 100,000 (95% CI 97–116 and 94–111, respectively; *P* > 0.05)] (6). The recently reported decreases in TIA are likely a result of improved control of risk factors, including hypertension, diabetes, dyslipidemia, and smoking (7). Because of known sex differences in risk factor profiles, a better understanding of temporal patterns in the prevalence of TIA risk factors by sex is necessary to reduce TIA incidence and mortality in both sexes.

The purpose of this study was to determine whether there were sex differences in the distribution of risk factors among a population of individuals with TIA from eastern China and provide further recommendations for stroke management and prevention in high-risk populations.

## Subjects and Methods

### Ethics Statement

The study was conducted according to the guidelines of the Helsinki Declaration. Ethical approval was obtained prior to the start of the study from the Ethics Committee of Shandong Provincial Hospital affiliated to Shandong University, China. Written informed consent was obtained from all participants.

### Study Population

The study population and proportion of the population to screen were determined according to specific criteria. First, provinces in China were selected based on accessibility and feasibility for long-term follow up of high-risk groups. Second, the proportion of the target population for screening in each region was determined based on the Sixth National Population Census of the urban and rural resident population for all persons >40 years of age. Proportional screening was undertaken according to the age distribution and sex ratio of the Sixth National Population Census of the population in each province. Further, based on the Census definition of a permanent resident population, we classified the population in a city divided into districts and the entire population in a city not divided into districts as urban residents, and the rest of the population as rural residents. Lastly, cluster sampling was used in 18 urban regions and 18 rural regions of Shandong province as a representative province of eastern China, characterized by a large population and rapid economic development.

The number of individuals screened within each region was no fewer than 6,000. We identified 231,289 (108,230 men and 123,059 women) permanent residents over 40 years old (date of birth between January 1, 1937 and December 31, 1971) in our screened regions who were eligible to participate in our study. Residents living for ≥6 months in an area were also included in our study, but recent migrants between urban and rural areas were not considered for inclusion. We selected 8,603 participants (3,390 men and 5,213 women) who had accurate data on a diagnosis of transient ischemic attack (TIA) in a medical institution above the township level. The prevalence of TIA was 3.1% in men and 4.2% in women.

### Data Collection

All data were collected using a nationally agreed question-naire. The survey questionnaire included: (1) basic information about the respondent(s), including age, gender, BMI, exercise status, community, education level and smoking history; (2) incidence or family history of hypertension, atrial fibrillation, diabetes grade, and stroke; (3) whether TIA was diagnosed by a medical institution above the township level, and whether there are symptoms of transient neurological impairment at the time of onset, including transient dizziness, visual rotation, limb weakness or numbness, language disorders, and coughing while drinking water. Duration, number of TIA, and time of the last TIA were also recorded. If the respondent could accurately provide a basis for the diagnosis of TIA in a medical institution above the township level, we diagnosed the patient as suffering from TIA.

### Assessment Criteria

This study was conducted to evaluate the use of symptom duration of 24 h or less as a segmentation point for the diagnosis of TIA. Previously in 2009, the American heart association and the American stroke association (ASA/AHA) had published new guidelines for TIA diagnosis. However, community epidemiology studies routinely use the histologic standard for diagnosis, and given the results of previous epidemiological studies on international comparability of data, we chose to adopt symptom duration of 24 h or less for the diagnosis of TIA. Patients with a previous TIA episode, irrespective of whether it was the first episode or a recurrence, were included in the diagnosis and patients with a simultaneous stroke episode were also categorized as TIA, regardless of whether the stroke occurred before or after the TIA.

RF1: hypertension, defined as a history of high blood pressure (≥140/90 mmHg) reported by the participant or the current use of antihypertensives. RF2: Atrial Fibrillation (AF) reported by the participant or indicated by electrocardiogram. RF3: diabetes mellitus defined by previous diagnosis, treatment with insulin/oral hypoglycemic medications, or a fasting plasma glucose level ≥126 mg/dL or glycosylated hemoglobin ≥6.5%. RF4: smoking defined by either the current or former practice of smoking. RF5: lack of physical exercise defined as physical exercise <3 times a week and each <30 min in duration (industrial and agricultural labor was considered as physical exercise). RF6: overweight, defined as a body mass index ≥25 kg/m2. RF7: self-reported family history of stroke.

### Statistical Analysis

Descriptive characteristics of study subjects according to differences in sex or residence are reported as percentages for categorical variables and mean ± standard deviation for continuous variables. Student's *t*-test was performed to assess differences in age, whereas Chi-square test was used to compare frequencies of education level and TIA risk assessments between men and women. Binary logistic regression modeling was used to estimate associations between sex and hypertension, diabetes mellitus, atrial fibrillation, smoking, lack of exercise, overweight, and family history of stroke after adjusting for age and degree of education. Results are expressed as multivariable-adjusted odds ratios (ORs) and 95% CI. A two-sided *P*-value <0.05 was considered statistically significant. All data analyses were performed using SPSS 25.0.

## Results

Descriptive characteristics of our study population are shown in Table 1. Our analysis cohort consisted of 8,603 individuals with TIA from Shandong province in eastern China, of which 3,390 (39.4%) were male and 5,213 (60.6%) were female with an average age of 61.9 ± 9.9 and 60.75 ± 9.9 years, respectively. We found that the mean age difference of TIA occurrence and education level between males and females were significantly different (*P* < 0.001), although there was no evidence of a sex difference in urban-rural distribution (*P* = 0.063). The age of TIA occurrence and education level in men were higher compared with those of women in our cohort.

**Table 1:** Descriptive characteristics of the study cohort.

**Risk factors**	**Male**	**Female**	**t/x^**2**^**	P^*^
Age	61.9 ± 9.90	60.75 ± 9.93	5.226	0.000
Education level			243.729	0.000
≤Primary school	1,866 (55.0%)	3,722 (71.4%)		
Middle school	1,043 (30.8%)	1,040 (20.0)		
High school	360 (10.6%)	350 (6.7%)		
University	118 (3.5%)	100 (1.9%)		
Master's degree	3 (0.1%)	1 (0%)		
Group			3.448	0.063
Urban	1,660 (49.0%)	2,446 (46.9%)		
Rural	1,730 (31.0%)	2,767 (53.1%)		

*Values are mean (SD) or percentages.*

* *Student's t-test was used for comparison of mean values and x^2^ test was used for comparison of proportions*.

Next, the distribution of different TIA risk factors were analyzed between men and women (Tables 2, 3). As shown in Table 2, we found that hypertension was the most common risk factor, with a higher prevalence in men (58.8%) compared with women (55.5%). Further, smoking was the risk factor with the greatest difference between sexes, in which 41.3% of men smoked compared with 4.2% of women. With the exception of exercise deficiency (*P* = 0.385), we also found evidence of increased rates of other TIA risk factors namely, hypertension and family history of stroke (58.8 and 22.3%, respectively) in men compared with women (55.5 and 20.0%, respectively). In contrast, the incidence of atrial fibrillation, diabetes, and overweight was higher in women (16.4, 13.2, and 35.7%, respectively) compared with that found in men (14.7, 11.1, and 29.5%, respectively). Using multivariate analysis (Table 3), we found significant differences between men and women in the distribution of four TIA risk factors specifically, hypertension (OR = 0.865, 95% CI 0.791–0.984), atrial fibrillation (OR = 1.230, 95% CI 1.087–1.392), diabetes (OR = 0.786, 95% CI 0.684–0.901), and overweight (OR = 0.754, 95% CI 0.686–0.830). In addition, among the variables included in logistic regression, we found that hypertension and smoking were more common in men.

**Table 2:** Risk factors for transient ischemic attack (TIA) in men and women.

**Risk factors**	**Male**	**Female**	**t/x^**2**^**	***P***
Hypertension	1,993 (58.8%)	2,893 (55.5%)	9.087	0.003
Atrial fibrillation	498 (14.7%)	856 (16.4%)	4.637	0.031
Smoking	1,400 (41.3%)	220 (4.2%)	847.593	0.000
Diabetes	373 (11.1%)	686 (13.2%)	8.358	0.004
Sport lack	972 (27.2%)	1,462 (28.0%)	0.755	0.385
Overweight	1,001 (29.5%)	1,863 (35.7%)	35.666	0.000
Family history of stroke	757 (22.3%)	1044 (20.0%)	6.5855	0.01

**Table 3:** Associations between risk factors for transient ischemic attack and sex in our study cohort.

**Risk factors**	***P***	**OR**	**95%CI**
Hypertension	0.002	0.865	0.791–0.984
Atrial fibrillation	0.001	1.230	1.087–1.392
Smoking	0.000	17.064	14.614–19.924
Diabetes	0.001	0.786	0.685–0.901
Overweight	0.000	0.754	0.686–0.830
Family history of stroke	0.079	1.103	0.989–1.231

We also studied gender differences in the prevalence of risk factors in the non-TIA population (Table 4) and compared it with the TIA population (Table 5). In addition to smoking (32.2 vs. 2.2%), hypertension (19.7 vs. 22.5%), atrial fibrillation (2.5 vs. 3.6%), diabetes (4.0 vs. 5.3%), reduced physical activity (13.6 vs. 16.7%), and prevalence of overweight (20.5 vs. 27.5%), a family history of stroke (6.8 vs. 7.4%) was more common in women than in men. Further, analysis of combined data provided in Tables 4, 5 showed that the prevalence of risk factors, in both men and women, was higher in the TIA group than in the non-TIA group. However, family history of stroke and hypertension were more common in men than women in the TIA group, whereas a greater number of women were present in the TIA group.

**Table 4:** Risk factors for non-TIA in men and women.

**Risk factors**	**Male**	**Female**
Hypertension	20,683 (19.7%)	26,491 (22.5%)
Atrial Fibrillation	2,610 (2.5%)	4,270 (3.6%)
Smoking	33,757 (32.2%)	2,627 (2.2%)
Diabetes	4,225 (4.0%)	6,261 (5.3%)
Sport lack	14,287 (13.6%)	19,626 (16.7%)
Overweight	21,449 (20.5%)	32,420 (27.5%)
Family history of stroke	7,173 (6.8%)	8,750 (7.4%)

**Table 5:** Risk factors in the non-TIA population compared with the TIA population.

	**TIA**		**Non-TIA**	
**Prevalence rates**	**Male**	**Female**	**Male**	**Female**
Hypertension	58.8%	55.5%	19.7%	22.5%
Atrial Fibrillation	14.7%	16.4%	2.5%	3.6%
Smoking	41.3%	4.2%	32.2%	2.2%
Diabetes	11.1%	13.2%	4.0%	5.3%
Sport lack	27.2%	28.0%	13.6%	16.7%
Overweight	29.5%	35.7%	20.5%	27.5%
Family history of stroke	22.3%	20.0%	6.8%	7.4%

## Discussion

After adjusting for multiple cofactors, we found significant differences in the distribution of a number of risk factors for TIA namely, hypertension, atrial fibrillation, smoking, overweight, and diabetes, between men and women. In the current study, the mean age of males was 61.9 years compared to 60.7 years in women. The prevalence of TIA in men and women was 3.1 and 4.2%, respectively, with a higher prevalence in women than in men. Concurrently, previous studies have also reported that the lower incidence of stroke in women disappeared with increasing age (8). Women over 80 have higher mortality and poorer functional outcomes compared to age-matched men after stroke. Previous studies have reported that women have a longer life expectancy than men, and that women are 4–6 years older than men at the onset of stroke, which may account for their higher disability rate and poorer prognosis (9, 10). TIA prevalence rates vary, depending on the age distribution of the study population. Other studies have found that a prevalence of TIA in men of 2.7% for 65 to 69 years of age and 3.6% for 75 to 79 years of age. For women, TIA prevalence was 1.6% for 65 to 69 years of age and 4.1% for 75 to 79 years of age (11). Similarly, a population-based study in Sweden found stroke incidence to be lower among women than men at ages 55–64 years; however, this association was reversed among individuals aged 75–85 years as incidence was higher among women than men (12). Other studies have reported contrasting results, with higher stroke risk seen in men compared to women, which then persisted after midlife or diminished, but never reversed with age (13). Our findings from the current study differ from those previously reported, which may be accounted for by population or ethnicity differences. In contrast to these earlier studies that tested other populations, our study investigated a Chinese cohort. In studies of gender differences in pre-hospital and hospitalization delays, women arrived both later than (14) and earlier than men (15). One study found that 40% of women arrived within 3 h of onset compared with 47% of men (14). However, there was no conclusive evidence on clinically important differences in pre-hospital delay between women and men.

Although stroke mortality and case fatality have declined in eastern China, improvements in standard of living has resulted in poorer control of risk factors, such as hypertension, diabetes mellitus, obesity, and smoking. In the current study, hypertension were found to be the most common risk factor in the TIA population. The incidence of hypertension was 58.8% in males and 55.5% in females and there was a significant difference between males and females in the distribution of Chinese Population (OR = 0.8650, 95% CI 0.791–0.984). In the non-TIA population, the incidence of hypertension was 19.7% in men and 22.5% in women. Hypertension is the most common modifiable stroke risk factor known to differ in prevalence, rate of control, and degree of associated stroke risk between women and men (16). Studies have shown that blood pressure is higher in men than women of similar ages (17); however, the import of sex differences in the prevalence of hypertension may be more complicated. A different study reported that prevalence is lower in women compared with men <60 years of age, but higher in women after that time point (18). Further, women in older age groups are less likely to control their hypertension compared with men (19). Whereas, data from UK found that women were less likely to be discharged on dual antiplatelet therapy, angiotensin-converting enzyme inhibitors, or statins (20). We hypothesized that women with low adherence to medication may be at greater risk for TIA in the presence of hypertension. A recent Chinese study found evidence of a strong association between hypertension and stroke risk in older women (OR = 6.73, 95% CI 2.14–21.15) compared with older men (OR = 3.18, 95% CI 1.65–6.14) (21). First, biological, genetic, or hormonal mechanisms linking hypertension to vascular dysfunction and disease may differ between the sexes (22). It is also possible that there are sex differences in hypertension treatment and adherence, which leads to increased stroke risk for women.

In our study, we also found that smoking (OR = 17.064, 95% CI 14.614–19.924) was more frequent in men. Among non-TIA subjects, men were more likely to smoke than women (32.2 vs. 2.2%) Whereas atrial fibrillation (OR = 1.230, 95% CI 1.087-1.392), diabetes (OR = 0.786, 95% CI 0.685–0.901), and overweight (OR = 0.754, 95% CI 0.686–0.830) were more frequent in women. These findings are consistent with those of previous studies that reported unhealthy lifestyle habits, such as smoking, drinking, and eating a diet high in salt and fat, were more pronounced in men (23). Impaired endogenous fibrinolysis and reduced blood flow in the brain attributable to vasoconstriction by smoking are also associated with lacunar stroke development (24). Studies have shown that continuous constriction of blood vessels can lead to high blood pressure, and that smokers with high blood pressure have a 5-fold greater relative risk of stroke compared to smokers with normal blood pressure. This risk increases to 20-fold higher in smokers with high blood pressure (25). Generally, published evidence supports a similar association between hypertension and stroke risk for men and women, and a stronger association with diabetes and atrial fibrillation for women (26). Atrial fibrillation is more prevalent among elderly people, and female stroke patients are older at the time of their first TIA compared to men (27). Women with atrial fibrillation have nearly double the risk of stroke than men with the same risk factor (28). Use of warfarin anticoagulation for thrombo-embolic prophylaxis in patients with atrial fibrillation has been shown to be less common in women than in men (29). Several studies have also shown that women are generally at a higher risk for atrial fibrillation-related cardioembolic stroke (30). The underlying reason for the increased risk for stroke in women with atrial fibrillation is not fully known (31), although a study from the Swedish Stroke Register has shown that female patients with atrial fibrillation receive oral anticoagulant therapy less often than men (32). The higher prevalence of embolic strokes among women may to a large part explain their higher stroke severity.

Although both diabetes and metabolic syndrome (the clustering of obesity, abdominal obesity, dyslipidaemia, hypertension, and high plasma glucose) are recognized to increase the risk of ischaemic stroke in men and women (17). However, the study indicated that both risk factors, namely diabetes and metabolic syndrome affect women more (33). In our study, the prevalence of diabetes in the TIA group was 11.15% in men and 13.2% in women, while in the non-TIA group it was only 4.0% in men and 5.3% in women. The prevalence of overweight in men and women in the TIA group was 29.5 and 35.7%, respectively, and these values were 20.5 and 27.5%, respectively, in the non-TIA group. In both groups, compared to men, women showed a greater prevalence of diabetes and overweight population-based study in Denmark found that type 2 diabetes doubled the risk of stroke in men across all age groups, whereas in women the effect of diabetes on stroke risk was significantly higher (risk ratio [RR] 2.5–6.5) (34). A recent study found that metabolic syndrome doubled the risk of ischaemic stroke in women but had no effect in men (35). The increase in TIA can be associated with a rise of obesity and other metabolic-associated diseases as a result of significant lifestyle changes in the last several decades; a phenomenon that warrants our continued attention. Moreover, the association between TIA and body mass index (BMI) is well-established (36). A study from the United Kingdom found that a higher BMI is associated with increased risk of ischemic stroke in women (37). Our study found that, compared to men, a greater number of women reported physical inactivity, in both TIA and non-TIA groups. The protective effect of physical activity may be partly mediated by its role in reducing blood pressure and controlling other risk factors, such as diabetes and excess body weight (38). We propose that policy strategies to prevent TIA should include educating people on the importance of a healthy balanced lifestyle, especially among women.

In addition to these risk factors, other aspects such as hormonal status, pregnancy, and migraine headaches put women at greater risk than men. The results of this meta-analysis indicate that people with migraine are at an increased risk of ischemic stroke, and that this increased risk is only apparent in those who have migraines with aura and not in those with migraines without an aura (39). Additionally, these results suggest an ~2-fold higher risk of migraine among women compared to men, and factors that further increased their risk of ischemic stroke were age <45 years, smoking, and use of oral contraceptives. Among people with migraine, the risk of TIA appeared to be higher than their risk of ischemic stroke. Pre-menopausal estrogen in women may have a protective effect on the heart and cerebrovascular systems, but understandably, the risk of microemboli caused by migraines, birth control pills, menopause, and other risk factors is higher in women than in men (40). It is now well-recognized that estradiol plays a vital role in the ischemic brain. Animal models of experimental stroke have shown that estrogen (17-βestradiol) is a robust neuroprotective agent in both males and females (41). This may be one reason why the incidence of TIA in pre-menopausal women is lower than in men. But Among healthy post-menopausal women in the Women's Health Initiative (WHI) study, exogenous estrogen increased the risk of stroke (42). Some risk factors are specific to women of reproductive age. A recent meta-analysis concluded that oral contraceptives increased the risk of ischemic stroke by nearly 3-fold (RR 2.75,95%CI 2.24–3.38) (43). Pregnancy can lead to hemostatic changes, including increased levels of clotting factors, decreased levels of anticoagulants, and fibrinolytic activity, all of which can increase risk of thrombosis. However, the overall incidence of pregnancy-related strokes was low (44).

Regarding TIA risk factors as a whole, the underlying causes that lead to the observed sex-specific differences may be accounted for by several points. First, men are more likely to be affected by bad habits, which may lead to an earlier onset of hypertension compared to women. Second, underlying biological factors such as hormonal mechanisms or genetics may confer a higher susceptibility to diabetes or atrial fibrillation in women. Furthermore, in our cohort we found that women have a lower education level than men, which may contribute to a lack of public knowledge regarding TIA as well as poor drug compliance for the management of risk factors (45).

Major strengths of the current study are the large representative sample from eastern China, the identification of all relevant TIA risk factors, and a thorough statistical analysis in our investigation to determine whether there was evidence of sex differences in TIA risk factors in our cohort. However, there are several limitations that should be considered in the context of our findings. First, the scope of our study was restricted by the age of our participants. Individuals ≥40 but <80 years of age were included in our cohort, thereby preventing a more thorough analysis of age-related changes in the adult population. Second, there is a possible selection bias of the screening population because of geographic location, society, and structure of the questionnaire survey. Third, binary logistic regression modeling was implemented to analyze risk factors independently, without considering the possibility of interactions between them. In addition, we are unable to conduct further in-depth analyses of certain risk factors.

Nevertheless, despite the aforementioned limitations, our study significantly contributes to a better understanding of TIA populations in eastern China by identifying and characterizing sex differences in TIA risk factors. Our findings demonstrate that there are significant sex differences in most of the risk factors examined, with increased rates of atrial fibrillation, diabetes, and overweight found in women and increased prevalence of hypertension and smoking in men. A better understanding of the sex differences and underlying temporal patterns in the prevalence of stroke risk factors are necessary to develop strategies to reduce stroke incidence and mortality in both sexes, such as greater promotion of good living habits in men and increased awareness of stroke among women. An improved understanding of such differences are needed to ensure that TIA prevention strategies are effective for both women and men.

## Data Availability Statement

The raw data supporting the conclusions of this article will be made available by the authors, without undue reservation.

## Ethics Statement

The studies involving human participants were reviewed and approved by Shandong Provincial Hospital affiliated to Shandong First Medical University. The patients/participants provided their written informed consent to participate in this study.

## Author Contributions

WW: investigation, data curation, writing-reviewing, and editing. PS: investigation, conceptualization, methodology. FH: data curation and writing-original draft preparation. CQ: writing-reviewing, editing, and supervision. All authors listed have made a substantial, direct, and intellectual contribution to the work and approved it for publication.

## Conflict of Interest

The authors declare that the research was conducted in the absence of any commercial or financial relationships that could be construed as a potential conflict of interest.
